# MicroRNA levels in patients with chronic hepatitis B virus and HIV coinfection in a high-prevalence setting; KwaZulu-Natal, South Africa

**DOI:** 10.1186/s12879-024-09715-0

**Published:** 2024-08-16

**Authors:** Lulama Mthethwa, Raveen Parboosing, Nokukhanya Msomi

**Affiliations:** 1 Discipline of Virology, School of Laboratory Medicine and Medical Sciences, University of KwaZulu-Natal and National Health Laboratory Service, 800 Vusi Mzimela Road, Durban, 4058 South Africa; 2 Department of Virology, School of Pathology, Faculty of Health Sciences, University of the Witwatersrand, and National Health Laboratory Service (NHLS), Johannesburg, South Africa

**Keywords:** Chronic HBV, microRNA, HBV-HIV coinfection, Biomarkers, HBV viral load

## Abstract

**Background:**

Hepatitis B virus (HBV) and human immunodeficiency virus (HIV) co-infection are significant public health issues, despite the availability of an effective HBV vaccine for nearly three decades and the great progress that has been made in preventing and treating HIV. HBV and HIV both modulate micro-ribonucleic acids (microRNA) expression to support viral replication. The aim of this study was to describe the pattern of microRNA expression in patients coinfected with chronic HBV and HIV with varying disease severity, as indicated by Hepatitis B e antigen (HBeAg) status, HBV viral load, alanine transaminase (ALT) levels, and HIV viral load.

**Methods:**

Plasma microRNAs, specific to HBV, were measured by quantitative real-time polymerase chain reaction (qRT-PCR) in HBV and HIV-negative healthy controls (*n* = 23) and patients coinfected with chronic HBV-HIV (*n* = 50). MicroRNA expression levels were compared between patients with high vs low HBV viral load, HBeAg positive vs HBeAg negative, high vs low ALT levels, and high vs low HIV viral load. Additionally, HBV viral load, ALT levels, and HIV viral load were correlated with microRNA expression levels.

**Results:**

Significantly higher expression levels of selected microRNAs were observed in chronic HBV-HIV coinfected patients compared to healthy controls. Significantly higher expression levels of hsa-miR-122-5p, hsa-miR-192-5p, and hsa-miR-193b-3p were observed in patients with high HBV viral load compared with low HBV viral load patients, and the levels of these microRNAs were correlated with HBV viral load levels. Significantly higher levels of hsa-miR-15b-5p and hsa-miR-181b-5p were observed in HBeAg-negative patients.

**Conclusion:**

This study demonstrates the potential use of hsa-miR-15b-5p, hsa-miR-122-5p, hsa-miR-181b-5p, hsa-miR-192-5p and hsa-miR-193b-3p as additional diagnostic biomarkers in chronic HBV disease progression.

**Supplementary Information:**

The online version contains supplementary material available at 10.1186/s12879-024-09715-0.

## Introduction

Elimination of Hepatitis B virus (HBV) infection is still a global health challenge despite the availability of an effective prophylactic vaccine [1, 2]. HBV infection causes acute and chronic hepatitis and complications such as liver cirrhosis and hepatocellular carcinoma (HCC) [3, 4]. Even though there is limited information about HBV distribution and prevalence in some populations and regions, it is estimated to be the 7^th^ major cause of morbidity and death globally [5, 6]. HBV affects 296 million people globally and about 81 million of these are in sub-Saharan Africa, where 990 000 new infections occurred in 2019 and 80 000 individuals died from HBV infection-related complications [7]. In 2019, there were 1.1 million deaths globally due to viral hepatitis, of which 96% were due to HBV and Hepatitis C Virus (HCV), which is greater than HIV mortality [7]. Most of the viral hepatitis deaths were due to liver cirrhosis and HCC [7]. Approximately 3.5 million people are infected with HBV in South Africa [8, 9]. Globally, about 39 million people were living with HIV in 2022, and treatment options are more available worldwide for HIV compared to HBV or HCV [10]. About 2.7 million people are estimated to be co-infected with HBV and HIV worldwide. South Africa is an endemic setting for HBV and HIV infections [11, 12].

MicroRNAs’ role in HBV pathogenesis and prognosis has been previously investigated [13]. MicroRNAs are small non-coding, single-stranded RNAs that inhibit or degrade messenger ribonucleic acids (mRNAs) during post-transcriptional gene expression by binding to the 3’-untranslated region (3’-UTR) of the target mRNA [14–16]. They were first discovered in *Caenorhabditis elegans (C. elegans)* but have now been found in some viruses and in all multi-cellular eukaryotes [17]. Numerous studies have been conducted to investigate the role of microRNAs in cellular response regulation such as proliferation, protein synthesis, differentiation, energy production, and apoptosis [18, 19].

Through direct interactions with viruses or viral components, cellular microRNAs can negatively or positively affect viral replication. Certain microRNAs have been identified that directly target viral transcripts, affecting HBV replication such as hsa-miR-122 [20]. Altered expression of specific microRNAs has been associated with different stages of HBV infection and the progression of liver diseases, including cirrhosis and HCC [21]. MicroRNAs have been investigated for role as diagnostic biomarkers for monitoring chronic HBV disease progression [22]. Numerous studies have investigated the role of microRNAs in HBV mono-infection [23–26], However, there is a paucity of studies investigating microRNA profiles in chronic HBV-HIV coinfected individuals, especially in sub-Saharan Africa, which is endemic for both infections [27]. Therefore, we sought to describe the pattern of microRNA expression in chronic HBV and HIV coinfected patients with varying disease severity, as indicated by HBeAg status, HBV viral load, ALT levels, and HIV viral load.

## Materials and methods

### Study design and study samples

In this retrospective case–control study, microRNA profile was compared in samples stratified according to serological markers, viral load, and ALT levels. The 10 candidate microRNAs included hsa-miR-15b-5p, hsa-miR-20a-5p, hsa-miR-29a-3p, hsa-miR-122-5p, hsa-miR-125b-5p, hsa-miR-181b-5p, hsa-miR-192-5p, hsa-miR-193b-3p, hsa-miR-194-5p, and U6 snRNA which had been identified as being potentially specific for Hepatitis B viral infection on a miRTarBase database (http://miRTarBase.cuhk.edu.cn/). Samples stored at the Department of Virology, Inkosi Albert Luthuli Central Hospital (IALCH), Durban from 50 chronically infected with sub-genotype A1 HBV patients were used in this study. All samples were hepatitis B surface antigen (HBsAg)-positive for at least 6 months. HBV viral load, ALT, HBeAg, and HIV viral load results were available for these samples in the database from the previous study “Hepatitis B virus variants in HBV mono-infected and HIV/HBV co-infected samples in a high dual infection setting” and were downloaded anonymously together with demographic (age/gender) and other relevant clinical data (e.g., antiretroviral treatment, co-infections, co-morbidities, history of liver disease). Some patients were in the immune-active chronic HBV phase and the others were in the inactive chronic HBV clinical phase. This study cohort included 29 males (18–52 years) and 21 females (23–61 years). Samples were grouped according to HBeAg status (positive or negative), and ALT levels (≤ 35 or > 35 U/L); HBV viral load (≤ 1000 or > 1000 IU/ml); and HIV viral load (≤ 1000 or > 1000 IU/ml). The cut-off criteria for ALT and HBV viral load was based on that normal ALT levels for males are ≤ 35 U/L and ≤ 25 U/L for females therefore, ALT ≤ 35 U/L was taken as an inclusive cut-off value for low levels [28] and patients with HBV viral load of 1000 IU/ml identify as inactive carriers which are accompanied by normal or low ALT levels. 23 subjects, negative for the HBV core antigen, HBsAg and HIV stored at the Department of Virology, IALCH, Durban were used as the healthy control group. The control cohort included 7 males (18–47 years) and 16 females (13–62 years). This study was approved by University of KwaZulu-Natal Biomedical Research Ethics Committee (BREC 00002418/2021).

### RNA extraction and quantification

Plasma samples stored at –80 °C were thawed and mixed using a vortex mixer and RNA was extracted from 500 µl plasma using the NucliSens easyMAG system (Biomeriux, Marcy I’Etoile, France) following the manufacturer’s instructions. The ribonucleic acid (RNA) concentration was measured at an absorbance of 260 nm in a NanoDrop 1000 Spectrophotometer (Thermo Fisher Scientific, Wilmington, United States) using nuclease-free water as a blank. RNA extracts were stored at -80 °C.

### Reverse transcription (RT) or complementary DNA (cDNA) synthesis

The TaqMan microRNA reverse transcription kit (Applied Biosystems, Vilnius, Lithuania) and the custom RT primer pool (Applied Biosystems, Pleasanton, United States) were used to reverse-transcribe the total RNA to cDNA following the manufacturer’s instructions. The custom primer pool was prepared by combining 10 μl of each 5X RT stem-loop specific microRNA primer in 1.5 ml microcentrifuge, and 1X Tris–EDTA (TE) buffer was added to bring the final volume to 1000 μl. The RT reactions were performed in 8-strip tubes with 4 µl of RNA sample, 8 µl RT primer pool (containing 0.05X of each stem-loop microRNA-specific RT primer), 2 µl of 10X RT buffer, 0.4 µl of 2 mM of dNTPs with dTTP, 4 µl of 10 U/μl MultiScribe Reverse Transcriptase, 0.25 µl of 0.25 U/μl RNase inhibitor and 1.35 µl nuclease-free water. The stem-loop microRNA-specific RT primers were designed by Thermo Fisher Scientific, Pleasanton, United States. The reaction tubes were sealed, inverted to mix, and centrifuged for 10 s then incubated on ice for 5 min. The reaction tubes were placed on the ProFlex 96-well PCR System (Applied Biosystems, Foster City, United States) thermal cycler, following cycling parameters on Table S1. The RT products were used immediately for the cDNA preamplification step or stored at -20 °C for up to one week.

### cDNA preamplification

To ensure that there would be sufficient cDNA product to amplify, a preamplification step was added. The custom preamplification primer pool was prepared by combining 10 μl of each 20X TaqMan MicroRNA assay (Applied Biosystems, Pleasanton, United States) in a 1.5 ml microcentrifuge. The reaction components were prepared following Table S2-A and the cycling conditions were set following Table S2-B. The reaction tubes were removed from the thermal cycler, briefly centrifuged and 175 μl of 0.1X TE (pH 8.0) was added to dilute the preamplification reaction product. The reaction tubes were sealed, inverted to mix, and briefly centrifuged. The products were stored at -20 °C for use in real-time quantitative PCR for up to one week.

### Real-time quantitative PCR

Real-time quantitative PCR was used to assess the expression of microRNAs in preamplified products. A PCR reaction master mix was prepared for each microRNA assay containing 1 µl of 20X TaqMan microRNA Assay (Applied Biosystems, Pleasanton, United States), 10 µl of 2X TaqMan Universal Master mix II, No AmpErase UNG (Applied Biosystems, Vilnius, Lithuania) and 8.8 µl of nuclease-free water. The 20X TaqMan microRNA Assay contained specific reverse and forward primers and a TaqMan probe dye-labelled (FAM) (Applied Biosystems, Pleasanton, United States). The mixture was vortexed and centrifuged briefly to mix and collect contents properly. The 19 µl of the PCR reaction master mix was transferred to each well of the optical 96-well reaction plate, and 1 µl of the preamplified product was added. The plate was sealed with MicroAmp Optical Adhesive Film, vortexed briefly and centrifuged for 30 s to collect the contents to the bottom of the wells. The plate was placed on a QuantStudio 7 Flex Real-time PCR system (Applied Biosystems, Foster City, United States) for amplification. The cycling parameters were set following Table S3. Normalisation in microRNA expression analysis was performed using the U6 snRNA TaqMan microRNA assay (Applied Biosystems, Pleasanton, United States) as an endogenous control.

### Data analyses and statistical analyses

Quantitative PCR threshold cycles (Ct) represent the number of cycles required for the fluorescent signal to cross the threshold. Each target was quantified in duplicates per sample. Among the 10 microRNA panels chosen, the U6 snRNA microRNA was used as an endogenous control for the normalization of the nine targets in the panel. The amplification plots of microRNAs were analysed using Design and Analysis software version 2.6 (Applied Biosystems, Foster City, United States) for analysis of the Ct values. The cut-off Ct value for microRNAs expression was 35: Ct values ≥ 35 were regarded as undetectable and were substituted with a Ct value of 35 for further analysis [29]. The microRNA expression relative to small RNA U6 was reported as delta Ct (∆Ct), calculated by subtracting the average Ct of U6 RNA from the average Ct of microRNA target. To interpret the results, the relative change in expression was assessed using a comparative Ct method. Relative microRNA expression levels were presented as equal to 2^−∆∆Ct^ and delta-delta Ct (∆∆Ct) was calculated by subtracting ∆Ct average for the control group from ∆Ct of the target microRNA [30]. The normality tests were performed to assess whether the data was normally distributed or not using Kolmogorov–Smirnov test, the Shapiro–Wilk test, D'Agostino & Pearson test, and the Anderson Darling test (Table S4). The levels of expression for the microRNA targets were assessed in five subgroupings, chronic HBV vs healthy control samples, High vs low HBV viral load, HBeAg status (positive and negative), ALT levels, and high vs low HIV viral load. Expression levels of microRNAs in different groups were compared using an unpaired Mann–Whitney U test, and a Spearman’s correlation coefficient (rho) analysis was used to evaluate the association of clinical parameters with microRNAs. Possible confounding variables were tested using a linear regression model. The Benjamini–Hochberg FDR method was used to correct false discovery rate [31]. All statistical analyses were performed using GraphPad prism version 7.0 (GraphPad Software, San Diego, United States) and IBM SPSS Statistics version 28.0 (IBM SPSS, New York, United States). A two-tailed *p* value of < 0.05 was regarded as statistically significant.

## Results

### Sample characteristics

This study analysed samples from 50 patients with chronic HBV and 23 controls without serological evidence of HBV. Characteristics of female chronic HBV samples (42%), healthy female controls (69.6%), male chronic HBV samples (58%), and healthy male control samples (30.4%) are summarised in Table 1. A significant difference in age and gender distribution was observed between chronic HBV samples and healthy controls (Table 1). However, there was no significant difference in microRNA expressions between age groups and gender groups in chronic HBV and healthy control samples (Table S5).

**Table 1:** Characteristics of Chronic Hepatitis B samples and healthy controls

	**CHBV samples (*n* = 50)**	**Healthy controls (*n* = 23)**	***p*-value**
Age (years), median (IQR)	36.5 (12.25)	31 (21)	0.04
Gender n (%)			
Male	29 (58%)	7 (30.4%)	0.04
Female	21 (42%)	16 (69.6%)	
ALT (U/L), median (IQR)	35 (18)	Not Tested	
HBV DNA (log_10_ IU/ml), median (IQR)	2.19 (2.71)		
HBeAg Status n (%)			
Positive	37 (74%)		
Negative	13 (26%)		
HIV viral load (log_10_ IU/ml), median (IQR)	2.42 (3.38)		
CD4 + count (cells/mm3), median (IQR)	171 (255)		
HBV active ART, n (%)			
TDF + LAM	48 (96)		
TDF only	1 (2)		
LAM only	1 (2)		

*ALT* Alanine transaminase, *CHBV* Chronic hepatitis B virus, *HBeAg* Hepatitis B e antigen, *HBV DNA* Hepatitis B virus Deoxyribonucleic acid, *HIV* Human immunodeficiency virus, *IQR* Interquartile range, *IU/ml* International units per millilitre, *U/l* Units per litre, *ART* Antiretroviral treatment, *TDF* Tenofovir disoproxil fumarate, *LAM* Lamivudine

### Expression levels of microRNA panel in chronic HBV samples vs healthy controls

**Fig. 1:**
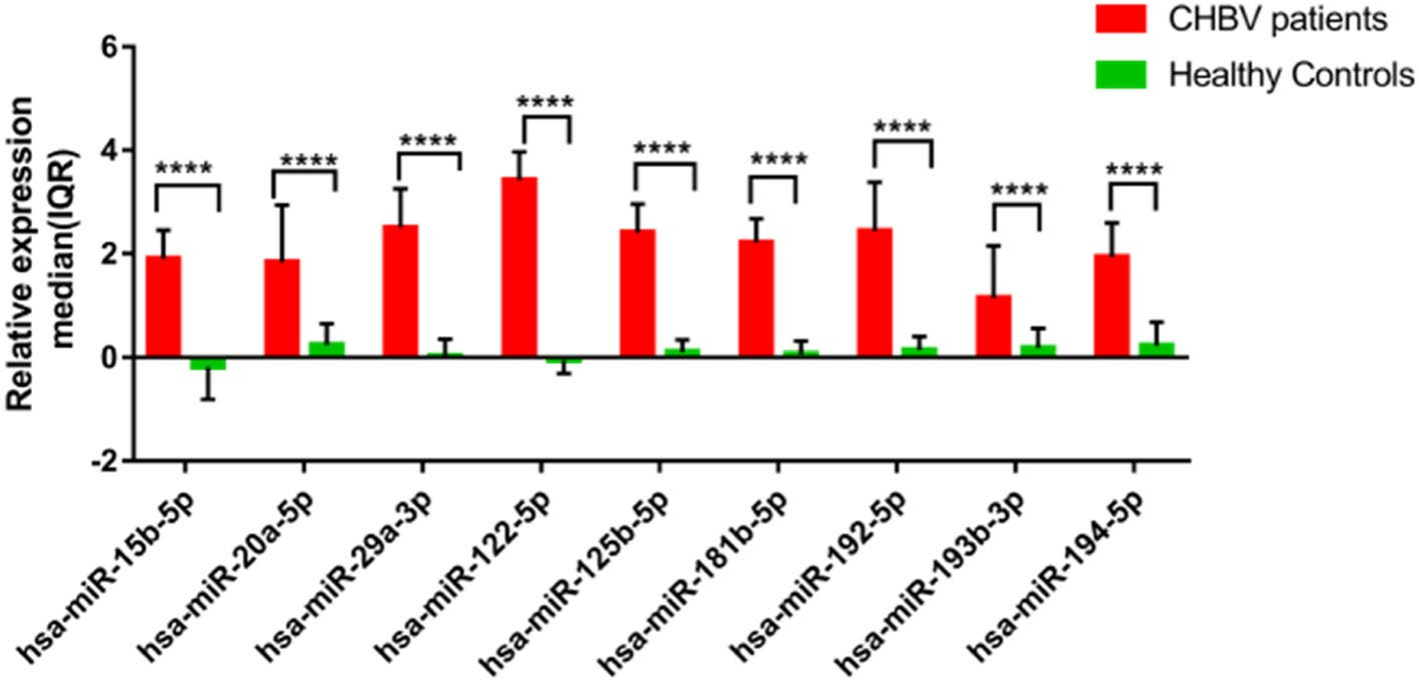
MicroRNA panel expression levels in chronic HBV samples compared to healthy controls. Relative microRNA level is shown as 2 ^ – (∆Ct of target microRNA– arithmetic mean of ∆Ct for the control group). Relative expressions are expressed as median and interquartile range. Statistical comparisons were performed using an unpaired Mann–Whitney U test. Significant differences are shown by an (*) system (**** *P* < 0.0001). CHBV, chronic hepatitis B virus; IQR, Interquartile range

Compared to healthy control groups, patients with chronic HBV infection showed significantly higher expression levels of all studied microRNAs (Fig. 1). The linear regression analysis revealed that age and gender did not influence the differences in the levels of microRNA expression, but these differences were due to HBsAg seropositivity (Table S7).

### Different microRNA expression signatures in chronic HBV samples with high vs low HBV DNA

**Fig. 2:**
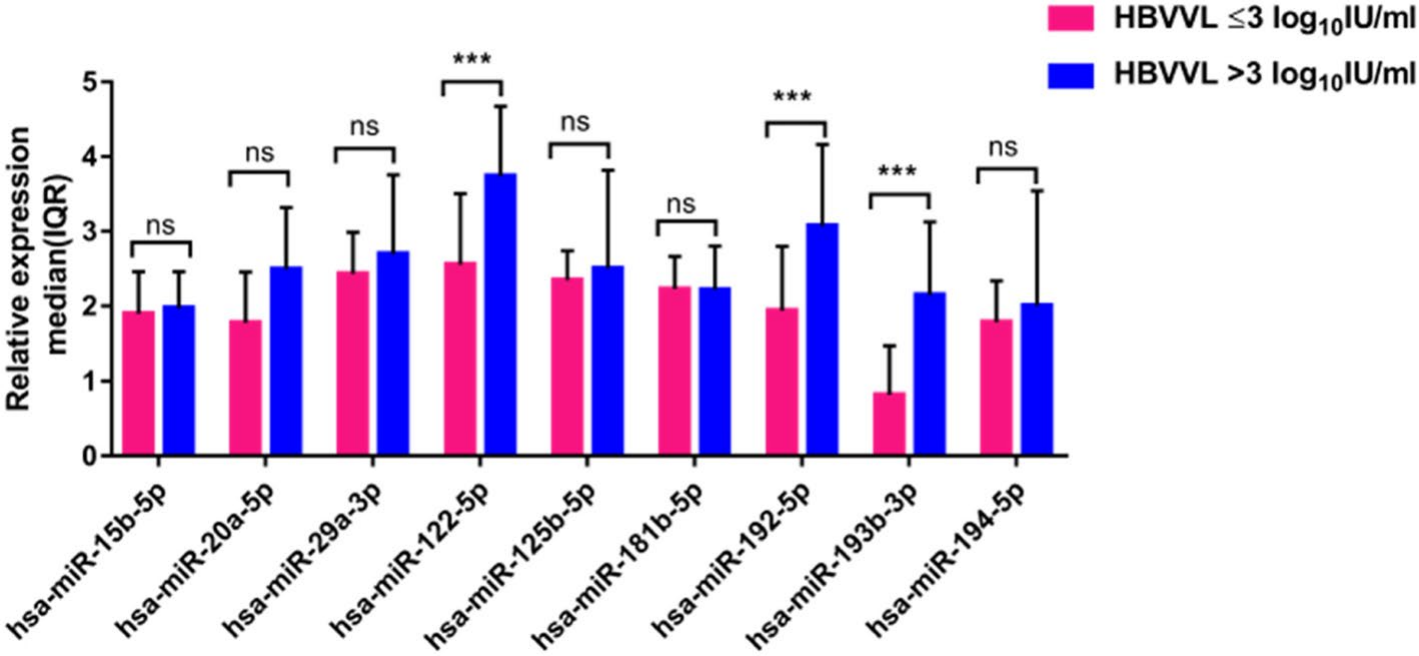
Different microRNA expression levels in chronic HBV samples with high and low HBV viral load. Relative microRNA level is shown as 2 ^ – (∆Ct of target microRNA– arithmetic mean of ∆Ct for the control group). Relative microRNA expressions are expressed as median and interquartile range. Statistical comparisons were performed using an unpaired Mann–Whitney U test. Significant differences are shown by an (*) system (*** *P* < 0.001, ns *P* > 0.05). HBVVL, Hepatitis B virus viral load; IQR, Interquartile range; IU/ml, international units per millilitre

**Fig. 3:**
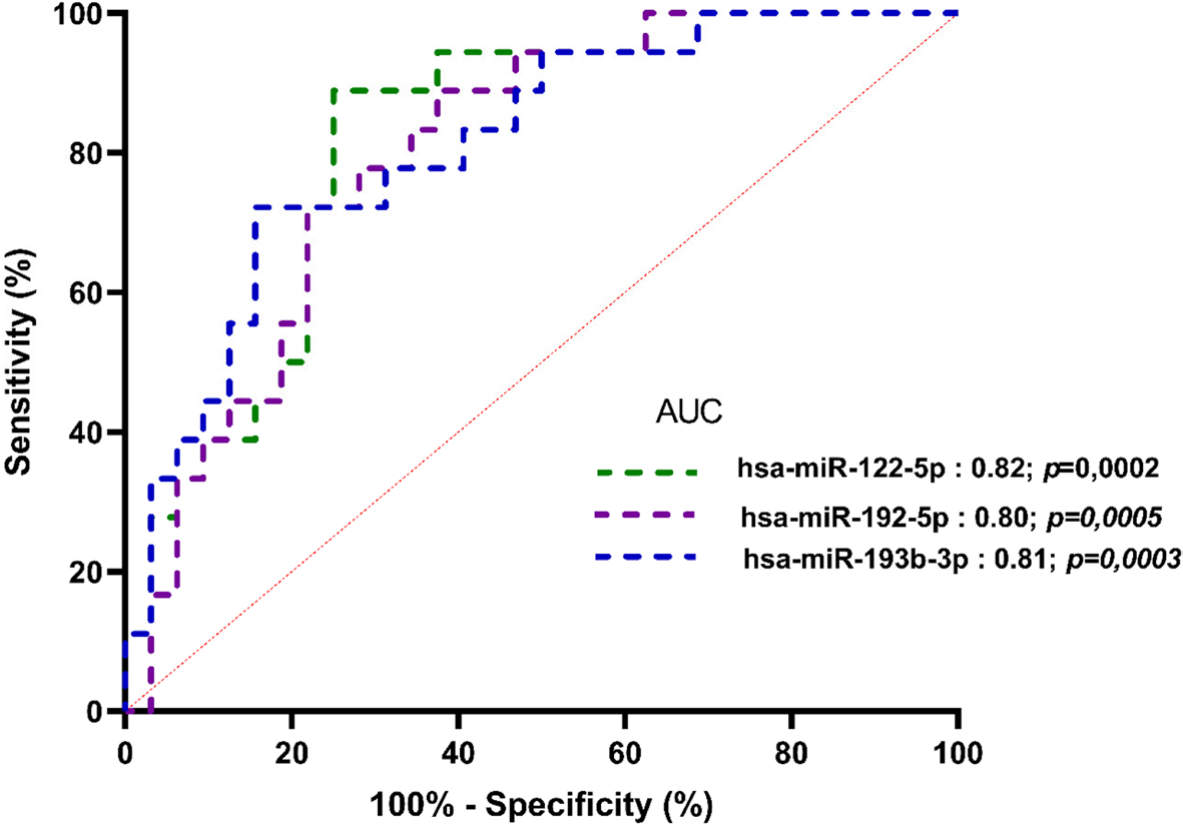
Differentiating power of hsa-miR-122-5p, hsa-miR-192-5p & hsa-miR-193b-3p in patients with low and high HBV viral load. Receiver operating characteristic curves and area under the curves (AUC) are presented for hsa-miR-122-5p, hsa-miR-192-5p & hsa-miR-193b-3p for differentiating patients with low HBV viral load from patients with high HBV viral load

Low HBV viral load (< 3 log_10_IU/ml) is whereby partial treatment response is generally considered and high HBV viral load (> 3 log_10_IU/ml) is whereby a virological breakthrough is defined [24, 28, 32, 33]. Samples with high HBV viral load had significantly higher median expression levels of specific microRNAs compared to samples with low HBV viral load – 3.74 vs 2.56 for hsa-miR-122-5p (*p* = 0.0001), 3.07 vs 1.94 for hsa-miR-192-5p (*p* = 0.0003) and 2.14 vs 0.82 for hsa-miR-193b-3p (*p* = 0.0002) (Table S6, Fig. 2). The expression levels of hsa-miR-20a-5p, hsa-miR-29a-5p, and hsa-miR-194-5p were slightly higher in patients with high HBV viral load compared to patients with low HBV viral load, but the difference was not statistically significant, while there was no significant difference in expression levels of microRNAs, hsa-miR-15b-5p, hsa-miR-125b-5p, and hsa-miR-181b-5p between high HBV and low HBV viral load samples. In samples with high HBV viral load hsa-miR-15b-5p had the lowest expression level followed by hsa-miR-194-5p (Table S6, Fig. 2), while hsa-miR-122-5p (median (IQR), 3.74 (1.18); *p* = 0.0001) had the highest expression level. A receiver operating characteristic (ROC) curve analysis was done to assess performance of hsa-miR-122-5p, hsa-miR-192-5p and hsa-miR-193b-3p in differentiating patients with low vs high HBV viral load (Fig. 3). The ROC curve revealed that the AUC was 0.82 (95% CI: 0.70 to 0.94, *p* = 0.0002) for hsa-miR-122-5p, 0.80 (95% CI: 0.68 to 0.92, *p* = 0.0005) for hsa-miR-192-5p and 0.81 (95% CI: 0.69 to 0.93, *P* = 0.0003) for hsa-miR-193b-3p for differentiating patients with low HBV viral load from patients with high HBV viral load.

### Expression levels of microRNAs associated with HBeAg status

**Fig. 4:**
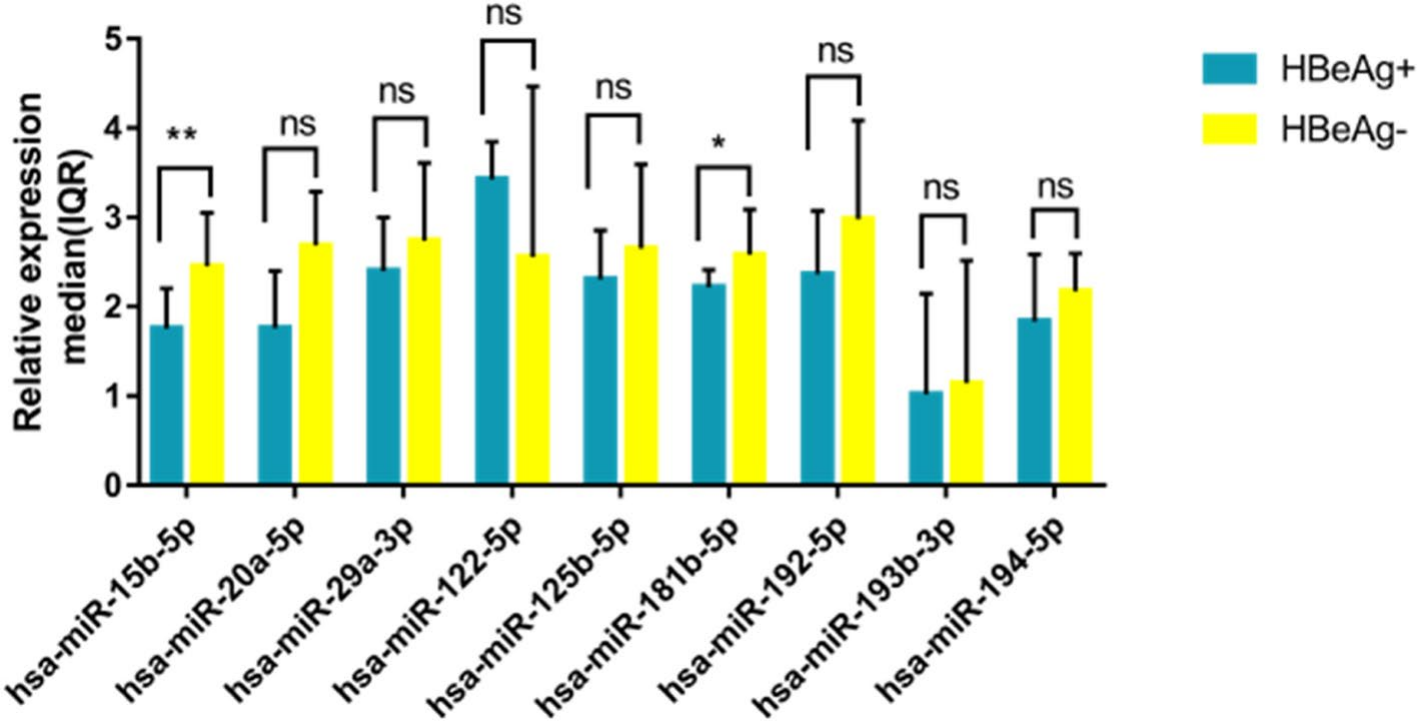
Plasma microRNA levels in chronic HBV samples from HBeAg ( +) and HBeAg (-) groups. Relative microRNA level is shown as 2 ^ – (∆Ct of target microRNA– arithmetic mean of ∆Ct for the control group). Relative microRNA expressions are expressed as median and interquartile range. Statistical comparisons were performed using an unpaired Mann–Whitney U test. Significant differences are shown by an (*) system (** *P* < 0.01, * *P* < 0.05, ns *P* > 0.05). HBeAg, Hepatitis B e Antigen; IQR, Interquartile range

Plasma microRNA levels were compared between chronic HBV samples who are positive and negative for the HBeAg. HBeAg-negative samples had significantly higher median levels of the following microRNAs compared to HBeAg-positive samples: hsa-miR-15b-5p (2.45 vs 1.76; *p* = 0.0054) and hsa-miR-181b-5p (2.58 vs 2.22; *p* = 0.03) (Table S6, Fig. 4). Slightly higher median expression levels were observed for hsa-miR-20a-5p, hsa-miR-29a-3p, hsa-miR-125b-5p, hsa-miR-192-5p, hsa-miR-193b-3p and hsa-miR-194-5p in HBeAg negative samples even though the differences were not significant compared to HBeAg positive samples (Table S6, Fig. 4). HBeAg positive samples had higher expression levels of hsa-miR-122-5p compared to HBeAg negative samples but the difference was not significant (Table S6, Fig. 4).

### Correlation of microRNA expression levels with markers of disease severity

**Fig. 5:**
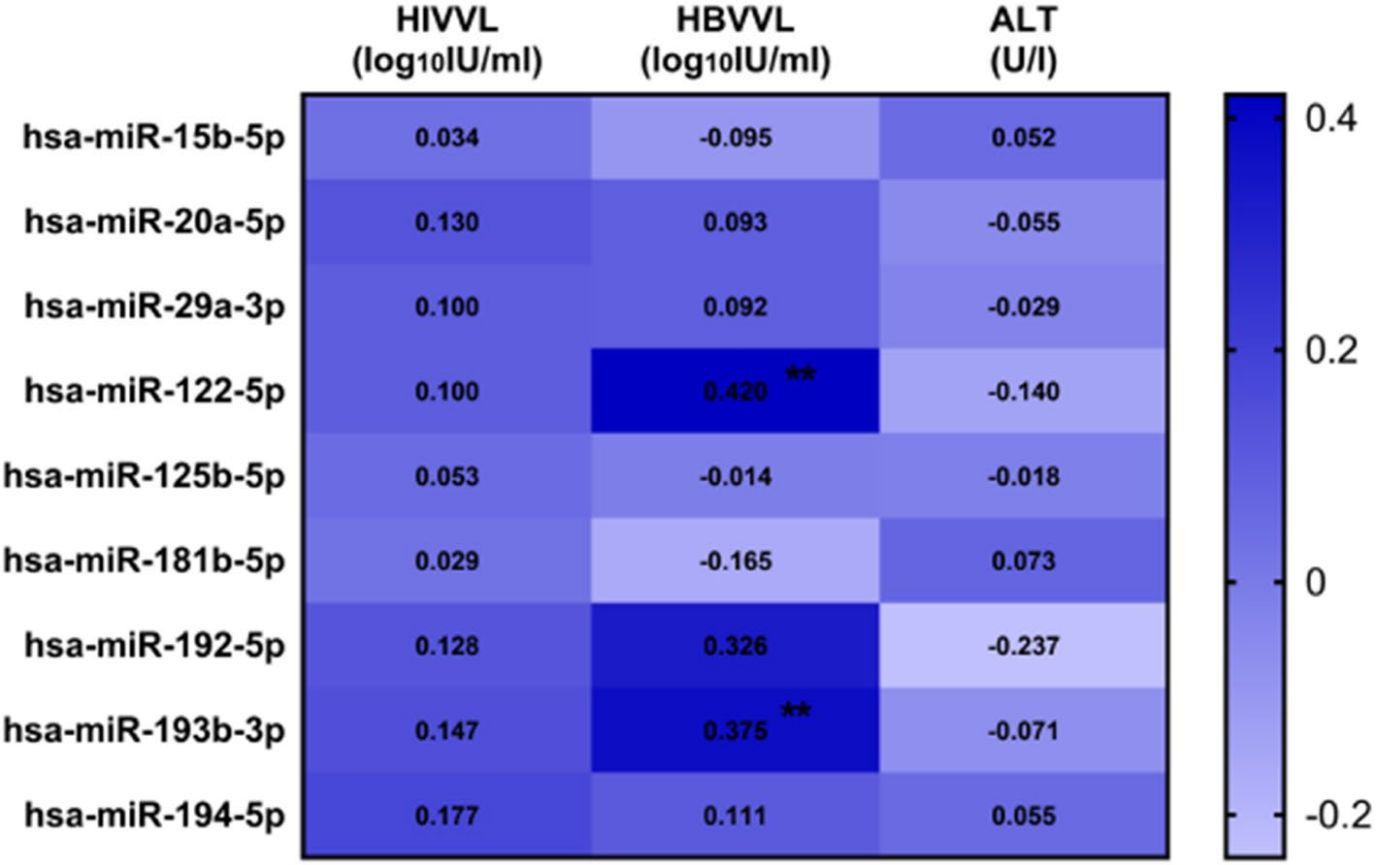
Heat map of the correlation of plasma relative microRNA level with HIV viral load, HBV viral load, and ALT level. Correlation coefficient (rho) was calculated using a Spearman correlation test. Significant relationships are shown by **P < 0.01. ALT, alanine transaminase; HBVVL, Hepatitis B virus viral load; HIVVL, human immunodeficiency virus viral load; IU/ml, international units per millilitre; U/l, Units per litre

A significant moderate positive correlation was observed between HBV viral load and hsa-miR-122-5p, hsa-miR-192-5p, and hsa-miR-193-3p (Fig. 5). To further confirm the association between HBV viral load and the abovementioned microRNA levels and to avoid any potential confounding effects of other variables such as HIV viral load, ALT levels, gender and age, a Benjamini–Hochberg correction test was performed. The significant positive correlation between HBV viral load with hsa-miR-122-5p and hsa-miR-193-3p was confirmed while the positive correlation between hsa-miR-192-5p and HBV viral load was found to be not significant. A weak positive correlation was observed between HBV viral load and hsa-miR-194-5p, and a weak negative correlation was observed between HBV viral load with hsa-miR15b-5p and hsa-miR-181b-5p, but these associations were not significant. No correlation was observed between HBV viral load and expression levels of hsa-miR-20a-5p, hsa-miR-29a-5p, and hsa-miR-125b-5p. No significant correlations were observed between our studied microRNAs and ALT levels, and HIV viral load. Our studied microRNAs were not able to differentiate elevated ALT levels from normal ALT levels as well as low vs high HIV viral load.

## Discussion and conclusion

Our study described the level of microRNA expression in samples from patients with chronic hepatitis B infection and furthermore compared microRNA expression in terms of various disease severity biomarkers (HBV viral load, HBeAg status, ALT levels, and HIV viral load). In our study, chronic HBV samples were compared with healthy samples and, all nine microRNA targets (hsa-miR-15b-5p, hsa-miR-20a-5p, hsa-miR-29a-3p, hsa-miR-122-5p, hsa-miR-125b-5p, hsa-miR-181b-5p, hsa-miR-192-5p, hsa-miR-193b-3p & hsa-miR-194-5p) revealed significantly higher levels in chronic HBV samples. The results from our study are congruent with the results from previous studies that investigated the expression pattern of the microRNAs in our panel in patients with chronic HBV [24, 34–37]. Chronic HBV infection is associated with significant morbidity and mortality. Chronic HBV infection is associated with a 15% to 40% risk of cirrhosis, HCC, and/or liver failure, as well as a 15% to 25% mortality risk from HBV-associated liver diseases [38]. It is essential to predict chronic HBV infection progression in patients so that antiviral therapy can be initiated at the right time. The current diagnostic markers for HBV infection can be used as indicators of specific infection phases, monitor the progress of infection and guide treatment decisions [39, 40]. Because of their non-invasive nature, serum or plasma microRNAs have attracted significant research attention as potential diagnostic and prognostic markers for chronic hepatitis B [41]. Several studies suggest that the use of microRNA panels in serum or plasma could improve the specificity of HBV diagnostics [13, 26, 42–44].

Patients with high HBV viral load expressed significantly higher levels of microRNA (hsa-miR-122-5p, hsa-miR-192-5p & hsa-miR-193b-3p), since HBV viral load levels are associated with disease progression, the levels of these microRNAs were also shown to be positively correlated with HBV viral load, these results suggest the potential use of these microRNAs as additional biomarkers for chronic hepatitis B disease progression. MicroRNAs may contribute to defining the phase of chronic hepatitis B infection by being involved in modulating the host immune response or contributing to inflammation and immune activation and/or assist in regulating viral gene expression and host immune responses, and treatment indication and may even allow assessment of antiviral therapy effectiveness [45]. Li et al*.* (2016) studied the expression levels of hsa-miR-125b-5p, and hsa-miR-122-5p and patients with high HBV viral loads demonstrated high expression levels of these microRNAs compared to patients with low HBV viral loads. In addition, elevated levels of hsa-miR-125b-5p were observed in patients in the immune-tolerant phase and at different stages of chronic hepatitis B infection. Lower levels of hsa-miR-125b-5p were observed in immune-tolerant phase compared to immune-reactive phase patients, which indicated that microRNAs may not only be influenced by HBV replication but by other factors such as liver necroinflammation [46]. These results may be relevant in the potential use of hsa-miR-122-5p, hsa-miR-192-5p, and hsa-miR-193b-3p as additional biomarkers for chronic HBV disease progression in our clinical setting.

In South Africa, the HBV genotype A is predominant with subtype A1 occurring in about 97% of rural Africans [47–49]. Infection with HBV genotype A is associated with chronicity, low HBeAg-positivity, horizontal transmission, and increased liver damage [50]. In this study expression levels of hsa-miR-15b-5p and hsa-miR-181b-5p were found to be significantly higher in HBeAg negative samples, in contrast to our findings, a study by Yu et al. (2015) found significantly high expression levels of hsa-miR-181b-5p in HBeAg-positive patients. Several previous studies based on microRNA profiling showed high expression levels of our studied microRNAs in HBeAg-positive patients when compared to HBeAg-negative patients [34, 51, 52]. This discordance between the findings in the current study and the abovementioned previous studies can be explained by the difference in specific geographical HBV genotypes and our cohort has sub-genotype A1 chronic HBV, most of the previous studies were done in settings predominated with genotype B, C & E chronic HBV. Different HBV genotype carriers are prone to different clinical outcomes or severity of the infection [49]. The evidence suggests that HBV genotypes may affect HBV endemicity, mutation patterns in the core and pre-core promoter regions, HBeAg seroconversion rates, clinical outcomes, and treatment response, however, the biological characteristics responsible for these differences have not yet been established [53, 54]. A study investigating HBV infection persistence found high persistence of HBV infection in patients with sub-genotype A1 when compared to non-A genotypes [55]. In chronic HBV patients without HBeAg, due to a mutation in the pre-core or core promoter region of the genome, HBV has a naturally occurring mutant that does not produce HBeAg [56, 57]. HBeAg-negative chronic HBV individuals may remain at risk for liver-related complications, especially if there is significant liver fibrosis or cirrhosis [38]. Therefore, non-invasive methods to monitor disease progression are needed. Hsa-miR-15b-5p and hsa-miR-181b-5p may serve as potential markers in HBeAg-negative chronic HBV disease progression. The assumption is that most of the study participants would have been infected as children and remained for a long period in the immune-tolerant phase, which is mediated by HBeAg status. Our results suggest that microRNA expressions of hsa-miR-15b-5p and hsa-miR-181b-5p may be associated with HBeAg production in our clinical setting and can be potentially used as biomarkers of HBV replication. The association of these microRNAs with HBeAg-negative status while the panel has correlation with HBV viral load could be explained by the following possible mechanisms such as these microRNAs may be associated with immune control mechanisms, influencing viral replication and clearance in the absence of HBeAg. The hsa-miR-15b-5p and hsa-miR-181b-5p microRNAs may have additional roles in influencing the clinical presentation, progression, or treatment response in HBeAg-negative patients. HBeAg-negative patients may have differences in the severity of liver disease, fibrosis, or risk of complications. These microRNAs might capture specific aspects related to disease progression or severity that are not solely reflected in HBV DNA levels such as co-infection with HIV.

This study also investigated the microRNA expression levels in high vs low HIV viral load and high vs low ALT levels but no significant differences were observed in the respective groups (Figure S1, Figure S2). This study investigated potential relationships between circulating microRNAs and HBV viral load, HIV viral load, as well as ALT levels, and a significant positive correlation was found between hsa-miR-122-5p and HBV viral load as well as between hsa-miR-192-5p and HBV viral load. We have similar results to previous studies that found hsa-miR-122-5p and hsa-miR-192-5p expression levels to be significantly correlated with HBV viral load [25, 34, 36]. The studies by van der Ree et al. (2017) and Wu et al. (2019) were conducted on patients that were undergoing antiviral treatment and the study by Winther et al. (2013) was conducted on patients’ mono-infected with chronic HBV. Our study was able to confirm these findings in samples with chronic HBV-HIV coinfection. According to these results, HBV viral replication may be influenced by miR-122-5p and miR-192-5p which could be assessed using cell culture, functional studies and other methods.

Other studies reported a significant negative correlation between HIV viral load and hsa-miR-29a-5p expression levels [37, 58, 59]. Our results did not show the potential role that our studied microRNAs may play in viral replication, and their potential to be utilized as a prognostic marker for HIV disease progression. Therefore, there is a need for more similar studies that will validate our findings or reveal the potential relationship that may exist between HIV viral load and our studied microRNAs. Our study showed no significant correlation between ALT levels and expression levels of our studied microRNAs. Wu et al. (2019) reported a negative correlation between hsa-miR-122-5p levels and ALT, however, another study found a positive correlation between ALT and hsa-miR-122-5p. In agreement with our findings, other studies found no correlation between hsa-miR-122-5p, hsa-miR-20a-5p, and hsa-miR181b-5p and ALT levels.

This is one of the few studies investigating microRNA profiling in a South African setting, a country where HBV genotype A1 predominates [60]. Hence, there is a need for more studies to be conducted in this setting to understand the role of microRNAs in the pathogenesis of chronic HBV infection. This study was able to show that the selected panel of microRNAs can differentiate between healthy control samples and chronic HBV samples, and that hsa-miR-122-5p, hsa-miR-192-5p & hsa-miR-193b-3p can potentially be used to differentiate between low versus high HBV viral load samples with over 80% accuracy which were supported by previous studies. However, further studies are required in this clinical setting to validate our findings. This study could not differentiate between low versus high ALT levels using microRNA profiling, which was supported by previous studies, therefore more future studies should investigate the association of microRNA expression with ALT levels, since ALT is considered to be a marker of active disease. The association of HBeAg status with specific microRNAs should be validated by more studies in our clinical setting to better understand the role of chronic HBV sub-genotype A1 on HBeAg seropositivity and disease progression and the role that microRNAs may play in viral replication. This study was able to correlate the hsa-miR-122-5p and hsa-miR-192-5p expression levels with HBV viral load which measures the virus titre in the bloodstream implying that these microRNAs have promising use as biomarkers for disease progression. Functional studies are needed to investigate the role of microRNAs in chronic HBV pathogenesis [61].

The sample size is one of the constraints of this study and the number of replicates used which were due to the costs of reagents used for testing. Future studies should increase the sample size and the number of replicates. Our microRNA profiles were not assessed according to clinical stage of HBV infection which should covered in further studies to better understand their role. Our studied microRNAs may have a function in the replication of HBV by targeting specific cellular factors or HBV transcripts. This microRNA panel can be utilised to differentiate patients with chronic HBV from healthy individuals in future studies, however, further validation studies are needed to establish the clinical utility of these microRNAs. Future studies should investigate the relationship between microRNAs and their presumed targets and HBV replication. It is possible that microRNAs may serve as markers for chronic HBV disease stages or prognostic markers for antiviral treatment response if they are found to play a direct or indirect role in regulating HBV replication. Furthermore, future research should include broader microRNA profiling to capture a more comprehensive picture of microRNA involvement in this intricate interplay between viruses and host responses.

Issues of chronic HBV infection such as HCC and cirrhosis often develop over decades, and HCC is often diagnosed much too late, leaving patients with poor prognoses and limited treatment options. For early detection of individuals at increased risk, sensitive and non-invasive methods are required that can detect subtle changes in the disease state. Through the monitoring of gene and microRNA expression in chronic HBV and liver disease, plasma or serum microRNA may be able to improve early detection. Therefore, our study was able to demonstrate the potential role of hsa-miR-15b-5p, hsa-miR-122-5p, hsa-miR-181b-5p, hsa-miR-192-5p and hsa-miR-193b-3p as biomarkers that could add to existing biomarkers with further studies, preferably prospective studies or retrospective where these markers are measured over time and associated/correlated to other clinical markers/signs for chronic HBV disease progression monitoring.

### Supplementary Information

Supplementary Material 1.

## Data Availability

Supplementary data can be found in the Supplementary Figures and Tables. Other data is available upon reasonable request from the corresponding author.

## Abbreviations

ALT: Alanine transaminase
BREC: Biomedical research ethics committee
cDNA: Complementary deoxyribonucleic acid
Ct: Threshold cycle
HBeAg: Hepatitis B e antigen
HBsAg: Hepatitis B surface antigen
HBV: Hepatitis B virus
HCC: Hepatocellular carcinoma
HCV: Hepatitis C virus
HIV: Human immunodeficiency virus
IALCH: Inkosi Albert Luthuli central hospital
MicroRNA: Micro-ribonucleic acids
mRNA: Messenger ribonucleic acid
qRT-PCR: Quantitative real-time polymerase chain reaction
RNA: Ribonucleic acid
RT: Reverse transcription
TE: Tris-EDTA
UTR: Untranslated region
