# Supplementary material for: MicroRNA levels in patients with chronic hepatitis B virus and HIV coinfection in a high-prevalence setting; KwaZulu-Natal, South Africa
Source: BMC Infect Dis. 2024 Aug 16;24:833. doi: 10.1186/s12879-024-09715-0 (PMC11328411; doi:10.1186/s12879-024-09715-0)
Supplement: Supplementary file 1 — Supplementary Material 1. [file 12879_2024_9715_MOESM1_ESM.docx]

**MicroRNA levels in patients with Chronic HBV and HIV coinfection in a high-prevalence setting; KwaZulu-Natal, South Africa**

**Supplementary Material**

**Table S1: Cycling parameters for reverse transcription**

| **Step** | **Time** | **Temperature (°C)** | **Cycles** |
| --- | --- | --- | --- |
| 1 | 30 minutes | 16 | 1 |
| 2 | 30 minutes | 42 |  |
| 3 | 5 minutes | 85 |  |
| 4 | ∞ | 4 |  |

**Table S2-A: cDNA preamplification reaction mixture**

| **Reagents** | **Volume per reaction (µl)** |
| --- | --- |
| RT reaction product | 2.5 |
| 2X TaqMan PreAmp master mix | 12.5 |
| preamplification primer pool | 3.75 |
| nuclease-free water | 6.25 |
| Total reaction | 25 |

**Table S2-B: cDNA preamplification reaction cycling parameters**

| **Step** | **Time** | **Temperature (°C)** | **Cycles** |
| --- | --- | --- | --- |
| Enzyme activation | 10 minutes | 95 | 1 |
| Annealing | 2 minutes | 55 | 1 |
| Extension | 2 minutes | 72 | 1 |
| Denaturation | 15 seconds | 95 | 12 |
| Annealing/extension | 4 minutes | 60 |  |
| Enzyme inactivation | 10 minutes | 99.9 | 1 |
| Hold | ∞ | 4 | ∞ |

**Table S3: Real time PCR amplification cycling parameters**

| **Step** | **Time** | **Temperature (°C)** | **Cycles** |
| --- | --- | --- | --- |
| Enzyme activation | 10 minutes | 95 | 1 |
| Denaturation | 15 seconds | 95 | 40 |
| Annealing/extension | 1 minute | 60 |  |

**Table S4: Tests for normal distribution of data**

| **Test for normal distribution** | **hsa-miR-15b-5p** | **hsa-miR-20a-5p** | **hsa-miR-29a-3p** | **hsa-miR-122-5p** | **hsa-miR-125b-5p** | **hsa-miR-181b-5p** | **hsa-miR-192-5p** | **hsa-miR-193b-3p** | **hsa-miR-194-5p** | **HIVVL** | **HBVVL** | **ALT** |
| --- | --- | --- | --- | --- | --- | --- | --- | --- | --- | --- | --- | --- |
|  |  |  |  |  |  |  |  |  |  |  |  |  |
| **Anderson-Darling test** |  |  |  |  |  |  |  |  |  |  |  |  |
| A2 | 0,77 | 1,5 | 1,6 | 0,58 | 1,8 | 1,4 | 0,47 | 0,51 | 1 | 10 | 17 | 0,45 |
| P value | 0,0436 | 0,0008 | 0,0003 | 0,1241 | 0,0001 | 0,0009 | 0,2366 | 0,1866 | 0,0085 | <0,0001 | <0,0001 | 0,2609 |
| Passed normality test (alpha=0.05) | No | No | No | Yes | No | No | Yes | Yes | No | No | No | Yes |
| P value summary | * | *** | *** | ns | *** | *** | ns | ns | ** | **** | **** | ns |
|  |  |  |  |  |  |  |  |  |  |  |  |  |
| **D'Agostino & Pearson test** |  |  |  |  |  |  |  |  |  |  |  |  |
| K2 | 4,2 | 5,8 | 26 | 5,5 | 17 | 14 | 9,2 | 2,5 | 2,8 | 38 | 63 | 7,7 |
| P value | 0,1244 | 0,0539 | <0,0001 | 0,0634 | 0,0002 | 0,0009 | 0,0103 | 0,2807 | 0,2465 | <0,0001 | <0,0001 | 0,0213 |
| Passed normality test (alpha=0.05) | Yes | Yes | No | Yes | No | No | No | Yes | Yes | No | No | No |
| P value summary | ns | ns | **** | ns | *** | *** | * | ns | ns | **** | **** | * |
|  |  |  |  |  |  |  |  |  |  |  |  |  |
| **Shapiro-Wilk test** |  |  |  |  |  |  |  |  |  |  |  |  |
| W | 0,96 | 0,93 | 0,88 | 0,96 | 0,9 | 0,92 | 0,96 | 0,98 | 0,95 | 0,53 | 0,29 | 0,96 |
| P value | 0,0968 | 0,0059 | 0,0001 | 0,0606 | 0,0004 | 0,0019 | 0,0816 | 0,3731 | 0,0466 | <0,0001 | <0,0001 | 0,1113 |
| Passed normality test (alpha=0.05) | Yes | No | No | Yes | No | No | Yes | Yes | No | No | No | Yes |
| P value summary | ns | ** | *** | ns | *** | ** | ns | ns | * | **** | **** | ns |
|  |  |  |  |  |  |  |  |  |  |  |  |  |
| **Kolmogorov-Smirnov test** |  |  |  |  |  |  |  |  |  |  |  |  |
| KS distance | 0,099 | 0,17 | 0,14 | 0,12 | 0,18 | 0,15 | 0,098 | 0,11 | 0,15 | 0,35 | 0,5 | 0,082 |
| P value | >0,1000 | 0,001 | 0,0129 | 0,0919 | 0,0002 | 0,0074 | >0,1000 | >0,1000 | 0,0051 | <0,0001 | <0,0001 | >0,1000 |
| Passed normality test (alpha=0.05) | Yes | No | No | Yes | No | No | Yes | Yes | No | No | No | Yes |
| P value summary | ns | ** | * | ns | *** | ** | ns | ns | ** | **** | **** | ns |
|  |  |  |  |  |  |  |  |  |  |  |  |  |
| Number of values | 50 | 50 | 50 | 50 | 50 | 50 | 50 | 50 | 50 | 50 | 50 | 50 |

ALT, alanine transaminase; HBVVL, hepatitis B virus viral load; HIV, human immunodeficiency virus viral load; A2, Anderson–Darling test statistic; K2, D'Agostino & Pearson test statistic; W, Shapiro-Wilk test statistic; KS, Kolmogorov-Smirnov test statistic

|  | **Healthy controls (n=23)** | | | | | | **CHBV samples (n=50)** | | | | | |
| --- | --- | --- | --- | --- | --- | --- | --- | --- | --- | --- | --- | --- |
|  | Age group | | | Gender | | | Age group | | | Gender | | |
|  | ≤32 | >32 | p-value | Female | Male | p-value | ≤37 | >37 | p-value | Female | Male | p-value |
| **Number** | 15 | 8 |  | 16 | 7 |  | 27 | 23 |  | 21 | 29 |  |
| **hsa-miR-15b-5p** | -0.18 (1.6) | 0.12 (1.71) | 0.83 | -0.41 (1.51) | 0.57 (1.85) | 0.77 | 1.76 (0.71) | 2.08 (1.07) | 0.16 | 1.77 (0.65) | 2.02 (1.09) | 0.35 |
| **hsa-miR-20a-5p** | 0.24 (0.84) | -0.08 (1.54) | 0.97 | -0.22 (1.28) | 0.32 (0.79) | 0.28 | 1.68 (1.16) | 1.99 (1.41) | 0.19 | 1.77 (0.89) | 1.88 (1.7) | 0.22 |
| **hsa-miR-29a-3p** | 0.03 (1.01) | 0.12 (0.91) | 0.43 | 0.02 (1.04) | 0.14 (0.51) | 0.82 | 2.49 (1.11) | 2.58 (0.95) | 0.83 | 2.51 (0.75) | 2.49 (1.49) | 0.69 |
| **hsa-miR-122-5p** | -0.05 (1.07) | -0.08 (1.40) | 0.47 | 0.02 (0.90) | -0.05 (0.34) | 0.41 | 3.42 (1.84) | 3.43 (1.68) | 0.89 | 3.44 (1.45) | 3.24 (1.98) | 0.85 |
| **hsa-miR-125b-5p** | 0.13 (0.81) | 0.04 (0.36) | 0.78 | 0.007 (0.79) | 0.13 (0.34) | 0.92 | 2.39 (0.47) | 2.43 (1.02) | >0.99 | 2.43 (0.57) | 2.39 (1.54) | 0.80 |
| **hsa-miR-181b-5p** | -0.05 (0.64) | 0.20 (1.36) | 0.24 | 0.01 (0.92) | 0.06 (0.69) | 0.82 | 2.22 (0.75) | 2.36 (1.02) | 0.25 | 2.22 (0.93) | 2.29 (1.26) | 0.45 |
| **hsa-miR-192-5p** | 0.13 (0.92) | 0.26 (0.75) | 0.39 | 0.13 (0.77) | 0.19 (0.62) | 0.72 | 2.46 (1.78) | 2.41 (1.48) | 0.63 | 2.46 (1.37) | 2.37 (2.04) | 0.89 |
| **hsa-miR-193b-3p** | 0.28 (1.03) | 0.17 (0.67) | 0.78 | 0.09 (1.43) | 0.49 (0.63) | 0.22 | 0.96 (1.60) | 1.40 (1.71) | 0.38 | 1.19 (1.18) | 0.98 (2.06) | 0.79 |
| **hsa-miR-194-5p** | -0.24 (1.62) | 0.27 (0.54) | 0.32 | -0.05 (1.25) | 0.28 (1.40) | 0.45 | 1.84 (1.10) | 2.01 (1.08) | 0.54 | 1.95 (0.91) | 1.87 (1.14) | 0.82 |

**Table S5: Comparisons of miRNA expression levels between different age groups and gender of the study participants**

CHBV, chronic hepatitis B virus

Relative expression levels (=2^- (∆Ct of target microRNA– arithmetic mean of ∆Ct for the control group)) are shown as median, the expression values are relative to the endogenous control expression level. The *p*-values are greater than 0.05.

**Table S6: Comparison of microRNA expression levels between different groupings of clinical** **biomarkers.**

|  | Study group | | | HBV Viral load (Log10 IU/ml) | | | HBeAg status | | | ALT Levels (U/l) | | | HIV viral load (Log10 IU/ml) | | |
| --- | --- | --- | --- | --- | --- | --- | --- | --- | --- | --- | --- | --- | --- | --- | --- |
|  | CHBV patients | Healthy controls | *P* value | ≤3 | >3 | *P* value | Positive | Negative | *P* value | ≤35 | >35 | *P* value | ≤3 | >3 | *P* value |
| Number | 50 | 23 |  | 32 | 18 |  | 37 | 13 |  | 26 | 24 |  | 30 | 20 |  |
| hsa-miR-15b-5p | 1.9 (0.91) | -0.18 (1.6) | **<0.0001** | 1.89 (0.94) | 1.97 (0.94) | 0.75 | 1.76 (0.69) | 2.45 (1) | **0.0054** | 1.89 (0.88) | 1.97 (1.04) | 0.41 | 1.9 (0.79) | 1.92 (0.91) | 0.75 |
| hsa-miR-20a-5p | 1.83 (1.38) | 0.24 (1.27) | **<0.0001** | 1.78 (0.94) | 2.49 (1.74) | 0.26 | 1.76 (0.9) | 2.69 (1.54) | 0.07 | 1.98 (1.05) | 1.77 (1.75) | >0.99 | 1.78 (1.17) | 1.93 (1.52) | 0.76 |
| hsa-miR-29a-3p | 2.51 (1.06) | 0.03 (0.76) | **<0.0001** | 2.43 (0.99) | 2.69 (1.42) | 0.08 | 2.40 (0.87) | 2.74 (1.14) | 0.07 | 2.55 (0.85) | 2.46 (1.19) | 0.89 | 2.50 (1.29) | 2.58 (1.19) | 0.79 |
| hsa-miR-122-5p | 3.42 (1.64) | -0.05 (0.53) | **<0.0001** | 2.56 (1.31) | 3.74 (1.18) | **0.0001** | 3.43 (1.49) | 2.56 (2.22) | 0.88 | 3.33 (1.61) | 3.47 (1.85) | 0.88 | 3.43 (1.92) | 3.26 (2.46) | 0.69 |
| hsa-miR-125b-5p | 2.41 (0.88) | 0.10 (0.6) | **<0.0001** | 2.35 (0.75) | 2.51 (1.53) | 0.19 | 2.31 (0.81) | 2.65 (1.35) | 0.14 | 2.46 (0.51) | 2.38 (1.78) | 0.75 | 2.51 (0.74) | 2.32 (0.71) | 0.48 |
| hsa-miR-181b-5p | 2.22 (0.85) | 0.06 (0.75) | **<0.0001** | 2.23 (1) | 2.22 (0.98) | 0.99 | 2.22 (0.85) | 2.58 (1.06) | **0.03** | 2.22 (1.06) | 2.22 (1.23) | 0.40 | 2.22 (1.20) | 2.22 (0.67) | 0.98 |
| hsa-miR-192-5p | 2.44 (1.64) | 0.13 (0.74) | **<0.0001** | 1.94 (1.34) | 3.07 (1.72) | **0.0003** | 2.37 (1.39) | 2.98 (2.31) | 0.22 | 2.46 (1.43) | 2.25 (1.9) | 0.39 | 2.35 (1.84) | 2.67 (1.52) | 0.69 |
| hsa-miR-193b-3p | 1.14 (1.46) | 0.18 (0.72) | **<0.0001** | 0.82 (1.08) | 2.14 (1.97) | **0.0002** | 1.02 (1.52) | 1.14 (1.62) | 0.49 | 1.04 (1.42) | 1.54 (1.84) | 0.73 | 1.06 (2.67) | 1.32 (1.69) | 0.31 |
| hsa-miR-194-5p | 1.93 (1.08) | 0.22 (1.21) | **<0.0001** | 1.78 (0.95) | 2.01 (1.75) | 0.08 | 1.84 (1.16) | 2.17 (0.91) | 0.20 | 1.85 (0.90) | 2.06 (1.37) | 0.16 | 1.83 (1.19) | 2.01 (1.08) | 0.46 |

ALT, alanine transaminase; CHBV, chronic hepatitis B virus; HBeAg, hepatitis B e antigen; HBV, hepatitis B virus; HIV, human immunodeficiency virus. Relative expression levels (=2^- (∆Ct of target microRNA– arithmetic mean of ∆Ct for the control group)) are shown as median (interquartile range (IQR)), the expression values are relative to the endogenous control expression level. The bold p-values are significant (<0.05)

**Table S7: Linear regression assessment of confounding effects in microRNA expression**

|  | | **CHBV + Controls** | |
| --- | --- | --- | --- |
| Model | | Standardized Coefficients  Beta | p-values |
| **1** | **hsa-miR-15b-5p** |  |  |
|  | AGE | 0.07 | 0.43 |
|  | Gender | -0.10 | 0.24 |
|  | HBsAg status | 0.70 | **<0.001** |
| **2** | **hsa-miR-20a-5p** |  |  |
|  | AGE | 0.04 | 0.66 |
|  | Gender | -0.15 | 0.06 |
|  | HBsAg status | 0.71 | **<0.001** |
| **3** | **hsa-miR-29a-3p** |  |  |
|  | AGE | 0.06 | 0.46 |
|  | Gender | -0.06 | 0.45 |
|  | HBsAg status | 0.74 | **<0.001** |
| **4** | **hsa-miR-122-5p** |  |  |
|  | AGE | 0.06 | 0.42 |
|  | Gender | 0.03 | 0.71 |
|  | HBsAg status | 0.82 | **<0.001** |
| **5** | **hsa-miR-125b-5p** |  |  |
|  | AGE | 0.04 | 0.63 |
|  | Gender | -0.07 | 0.36 |
|  | HBsAg status | 0.74 | **<0.001** |
| **6** | **hsa-miR-181b-5p** |  |  |
|  | AGE | 0.12 | 0.13 |
|  | Gender | -0.07 | 0.40 |
|  | HBsAg status | 0.71 | **<0.001** |
| **7** | **hsa-miR-192-5p** |  |  |
|  | AGE | -0.01 | 0.94 |
|  | Gender | -0.03 | 0.74 |
|  | HBsAg status | 0.72 | **<0.001** |
| **8** | **hsa-miR-193b-3p** |  |  |
|  | AGE | 0.02 | 0.82 |
|  | Gender | -0.05 | 0.65 |
|  | HBsAg status | 0.50 | **<0.001** |
| **9** | **hsa-miR-194-5p** |  |  |
|  | AGE | 0.08 | 0.33 |
|  | Gender | -0.03 | 0.75 |
|  | HBsAg status | 0.71 | **<0.001** |

CHBV, chronic hepatitis B virus; HBsAg, hepatitis B surface antigen

**
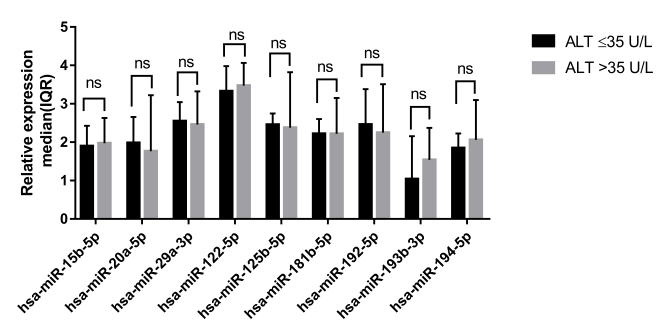
**

**Figure S1: MicroRNA expression levels comparison in different groupings of ALT levels.** Relative miRNA level is shown as 2 ^ – (∆Ct of target microRNA– arithmetic mean of ∆Ct for the control group). Relative microRNA expressions are expressed as median and interquartile range. Statistical comparisons were performed using an unpaired Mann-Whitney U test. Significant differences are shown by an (*) system (ns P≥0.05).

**
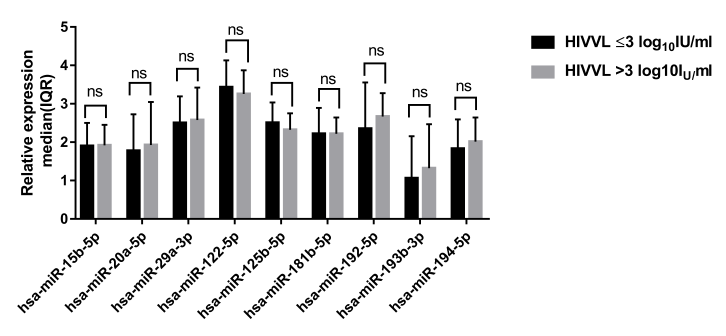
**

**Figure S2:** **MicroRNA expression signature comparison between samples with high vs low HIV viral load.** Relative miRNA level is shown as 2 ^ – (∆Ct of target microRNA– arithmetic mean of ∆Ct for the control group). Relative microRNA expressions are expressed as median and interquartile range. Statistical comparisons were performed using an unpaired Mann-Whitney U test. Significant differences are shown by an (*) system (ns P≥0.05)
